# Association of Early Renal Dysfunction with Lipid Profile Parameters among Hypertensives in Kazakhstan

**DOI:** 10.3390/diagnostics11050871

**Published:** 2021-05-12

**Authors:** Alma Nurtazina, Dana Kozhakhmetova, Daulet Dautov, Nurzhanat Khaidarova, Vijay Kumar Chattu

**Affiliations:** 1 Department of Epidemiology and Biostatistics, Semey Medical University, Semey 071400, Kazakhstan; 2 Department of Quality Assurance in Medical Education, Semey Medical University, Semey 071400, Kazakhstan; dana_ken@mail.ru; 3 Department of Propaedeutics of Internal Diseases, Kazakh Medical University, Almaty 050010, Kazakhstan; daudauda@gmail.com; 4 Department of Therapeutic Dentistry, Semey Medical University, Semey 071400, Kazakhstan; Nyrzhanat@mail.ru; 5 Department of Medicine, Temerty Faculty of Medicine, University of Toronto, Toronto, ON M5G 2C4, Canada; 6 Division of Occupational Medicine, Occupational Medicine Clinic, St. Michael’s Hospital, Toronto, ON M5C 2C5, Canada

**Keywords:** chronic kidney disease (CKD), early renal dysfunction, Apolipoprotein B, Apolipoprotein A1, low-density lipoprotein cholesterol (LDL-C), triglycerides (TG), high-density lipoprotein cholesterol (HDL-C), hypertension, estimated glomerular filtration rate (eGFR), Kazakhstan

## Abstract

Dyslipidemia plays an essential role in chronic kidney disease (CKD). The role of lipids and lipoproteins in the early pre-disease state of CKD in hypertensive patients is still unclear. The study aimed to evaluate the relationship between early renal dysfunction and lipid profile parameters among hypertensive patients in Kazakhstan. From April 2015 to December 2016, 800 Kazakh males and females with primary hypertension who met the inclusion criteria were included in this cross-sectional study. Data were collected on socio-demographics, lifestyle parameters, family history of cardiovascular disease, and hypertension. Additionally, Dietary Quality Score (DQS), anthropometric data, and blood pressure were recorded. Laboratory blood measurements included eGFR (estimated glomerular filtration rate), lipid profile parameters such as Apolipoprotein B, A1, HDL-C, LDL-C, and TG. We found a linear relationship between early renal dysfunction and LDL-C, Apolipoprotein B, and Apolipoprotein B/A1 ratio, which was in all cases negative and small (*r* = −0.27, −0.23 and −0.16, respectively). Apolipoprotein A1, HDL-C and TG have not revealed a linear relationship with GFR (*r* = −0.06, *r* = −0.06, and *ρ* = −0.045, respectively). The multicollinearity test restricted the linear model to Apolipoprotein B only. Further linear regression analysis confirmed an inverse significant linear association between eGFR and Apolipoprotein B. Age, DQS, and income appear to be positive confounding factors, significantly fitted the final model. ROC analysis had proven the predictive power of Apolipoprotein B in pre-CKD eGFR decline before and after adjustment for age, DQS and income (AUC = 0.62 and AUC = 0.77, respectively). For differentiating non-diabetic subjects with and without pre-CKD eGFR decrease, 1.05 g/L and 0.98 g/L are likely to be optimal cutoff points in males and females, respectively. These findings will help early prediction of renal dysfunction and contribute to a more accurate estimation of CVD risk.

## 1. Introduction

Chronic kidney disease (CKD) and dyslipidemia are mutually synergistic factors that seriously augment cardiovascular risk and CKD’s progression [1]. Premature atherosclerosis in CKD dramatically increases cardiovascular morbidity and end-stage renal disease (ESRD) rates. Over almost the three last decades, the global incidence of CKD increased by 89% and death by 98% [2]. The growing global burden and impairment caused by CKD necessitate early pre-case detection followed by preventive measures against CKD progression. Recent systematic reviews and meta-analyses suggest hypertension and obesity predict the onset of CKD in the general population [3,4]. CKD is associated with plasma lipoprotein metabolism disorders. Increased levels of triglycerides (TG), very low-density lipoproteins (VLDL), LDL-C, and Apolipoprotein B, versus HDL-C and Apolipoprotein A1 decrease, are common in CKD [5,6,7,8,9]. Hypertriglyceridemia’s onset refers to the early stages of CKD. Contemporaneously, a certain degree of controversy in this respect exists up to now [10,11]. Despite having a substantial body of scientific literature on the role of dyslipidemia in CKD patients, the predictive power of lipid profile parameters in the early pre-CKD condition and its relationship with non-CKD GFR decline remains unknown. As far as we know, no publications in this field are available. Therefore, we aimed to study the association of pre-CKD eGFR decline with lipid profile parameters in Kazakh hypertensives.

## 2. Materials and Methods

The design of the study is an analytical cross-sectional study. The current study is a part of a larger project previously published [12]. Participants included patients with primary hypertension (HT) recruited from sixteen primary care centers (PHCs) of Semey city in the East Kazakhstan region of the Republic of Kazakhstan from 6 April 2015 to 31 December 2016. All participants were interviewed, had a physical examination, and underwent laboratory testing before providing written informed consent to participate in the study. The study protocol was approved by the Ethics Committee at Semey Medical University (Ref No 0115РК1862, dated 18 February 2015). The final enrollment included 704 patients of Kazakh ethnicity, both men and women, aged 25 to 75 years with proven diagnosis of HT according to the clinical guidelines of the European Society of Hypertension and European Society of Cardiology (ESH/ESC) [13]. The exclusion criteria included previous stroke, myocardial infarction, existing diabetes mellitus, hypothyroidism (or taking thyroid hormones), thyrotoxicosis (or taking antithyroid agents), coronary artery disease, heart failure, any acute kidney condition, CKD, nephrotic syndrome, neoplasms, and mental illness. Pregnant and lactating women and those who took statins regularly less than six months before involvement in the study were also excluded from this research.

### 2.1. Sampling

A two-stage random sampling procedure was filled up. Initially, we selected 16 out of 40 PHCs at random in Semey city, East Kazakhstan. The sample frames for potential participants were assembled in every GP practice as per the inclusion criteria. A total of 50 subjects from every PHC were chosen by simple random sampling based on a random number generated by a computer in the second stage.

### 2.2. Variables

Initially, Apolipoprotein B, Apolipoprotein A1, Apolipoprotein B/A1 ratio, LDL-C, HDL-C, and TG were the primary risk factors. Apolipoprotein B has been accepted as the final main risk factor based on the results of correlation and multicollinearity analyses. The outcome of interest was an estimated glomerular filtration rate (eGFR). All other factors, such as gender, age, education, income, alcohol use, smoking, DQS, physical activity, family history of HT and CVD, plasma glucose, current obesity, and Metabolic Syndrome (MS), were considered as potential confounding factors.

### 2.3. Data Sources/Measurements

#### 2.3.1. Data Collection

All data for every study participant were recorded in an individual registration card (IRC) and assigned a unique code. After entering data into the electronic database, the next step was to double-check it. To warranty data confidentiality, only the project manager and the data entry specialist had access to the electronic database, which will be destroyed in five years.

All participants filled out structured questionnaires on socio-demographic indicators (gender, age, income, and education), lifestyle, and medical information (physical activity, smoking status, alcohol consumption, heredity for CVD and HT). Information such as full diagnosis, medications used was obtained from the medical records. A validated Dietary Quality Score (DQS) [14], translated into Russian, was used to assess dietary habits. Results of DQS were classified into three main groups: unhealthy dietary habits (1–4 points), intermediate (4–6 points), and healthy dietary habits (>6).

#### 2.3.2. Anthropometry Data and Blood Pressure Measurements

Height, weight, and waist circumference (WC) were measured by certified nursing staff using a stadiometer and weighing scale in the pre-doctor room of PHCs. Blood pressure (BP) was measured by the Korotkov method as per the ESH/ESC algorithm at rest in a sitting position. The WHO classification was used for categorizing BMI into three categories: <25 kg/m^2^ (normal weight), 25.0–29.9 kg/m^2^ (overweight) and >30 kg/m^2^ (obesity).

#### 2.3.3. Laboratory Data

Blood samples were taken in the morning by an intravenous puncture after a fasting period of 12 has a minimum. All biochemical and lipid profile parameters were determined by immunoturbidimetric method on analyzers “Cobas 6000”, biochemical module c501, Roche Diagnostics GmbH (registration certificate of the Republic of Kazakhstan No. РК-МТ-7№012668 dated 28 February 2014). Oral glucose tolerance test (GTT) was performed standardly after ingestion of 75 g oral glucose load and repeated glycemia measurement over two hours.

### 2.4. Diagnostic Criteria

#### 2.4.1. Hypertension

Essential Hypertension (HT) was diagnosed based on ESC recommendations [13]. A confirmed diagnosis of HT has been registered in medical records in all participants. All of them took antihypertensive agents regularly and were followed up by PHC general practitioners (GPs).

#### 2.4.2. Chronic Kidney Disease

According to KDIGO CKD Work Group [15], CKD was diagnosed based on the eGFR under 60 mL/min per 1.73 m^2^ and kidney structure abnormalities and function for more than three consequent months.

#### 2.4.3. eGFR Calculation

eGFR was calculated as per the Chronic Kidney Disease Epidemiology Collaboration (CKD-EPI) Group formula, which is considered more accurate at the eGFR above 60 mL/min per 1.73 m^2^ other formulas [16,17,18].

#### 2.4.4. Metabolic Syndrome

MS was determined based on IDF criteria [19], including abdominal obesity or BMI >30 kg/m^2^ accompanied by two of the following four factors: BPsyst > 130 mmHg or BPdiast > 85 mmHg, plasma of TG > 1.7 mmol/L, HDL-C < 1.03 mmol/L in men and <1.29 mmol/L in women, plasma glucose > 5.6 mmol/L.

#### 2.4.5. Obesity

Obesity was diagnosed based on WHO classification based on BMI [20].

### 2.5. Biases

A two-level random sampling was carried out to reduce selection bias and enhance study participants’ representativity regarding the reference population. To minimize the measurement error, height, weight, WC, and BP were measured in a standardized way by certified nurses of PHCs and trained research team staff; blood samples were taken and then tested in a single laboratory. Interviews were conducted in the same manner by specially trained research staff to reduce observer bias. The conditions, namely HT, CKD, MS, and obesity, were clearly defined based on the standard diagnostic criteria to prevent misclassification.

#### Study Size

**Figure 1:**
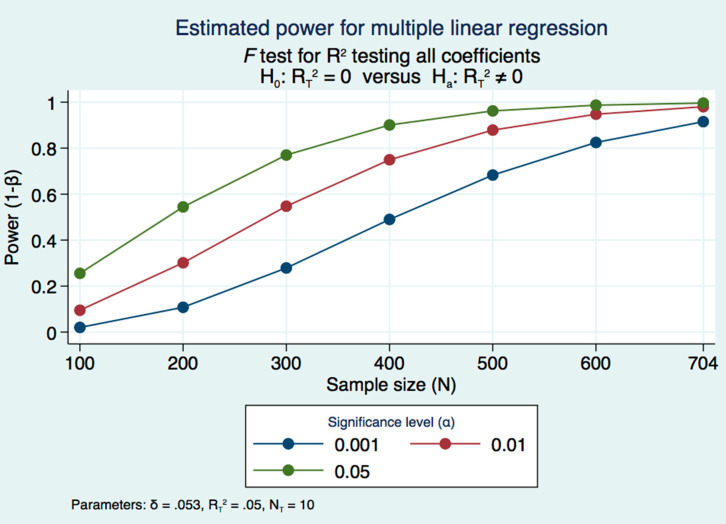
Estimated power for multiple linear regression. δ—effect size; R^2^—F-test for the joint effect of the coefficients of the full regression model; N—number of covariates/potential confounding factors; H_0_—the Null hypothesis; H_1_—the Alternative hypothesis.

Taking into account that our study is a part of the major project [12] with a sample size of 704 hypertensive patients and lack of data on pre-CKD in the available research literature, we decided to check whether this sample size has sufficient statistical power enough for finding an association between eGFR and lipid profile parameters comprising potential confounding factors. The power R-squared method for the multiple linear regression model has been applied. Given a sample size of 704 participants and assuming approximately 5% variance (R^2^ = 0.05) in the dependent variable, eGFR, we estimated statistical power for the multiple linear regression model with ten potential covariates on 0.05, 0.1, and 0.001 two-sided significance levels, 92%, 98%, and 99%, respectively (Figure 1).

### 2.6. Quantitative Variables

eGFR was employed as a quantitative continuous variable. Lipid profile parameters such as Apolipoprotein B, Apolipoprotein A1, Apolipoprotein B/A1, LDL-C, HDL-C, and TG were presented as quartiles Q1 to Q4. For testing a linear relationship with eGFR, all lipid profile parameters were used as continuous variables. Gender, family history status for HT and CVD, alcohol consumption status, and gym class attendance were presented as dichotomous variables. Smoking status was evaluated in three categories: not smoking, smoking, and quit. We divided all participants into five age strata, i.e., <39, 40–49, 50–59, 60–69, and >70 years old. DQS, education, income, and physical activity were employed as categorical, ordinal variables.

### 2.7. Statistical Methods

Statistical analysis was performed using Stata Statistical Software: Release 15, College Station, TX, USA: StataCorp LLC.

Categorical variables were presented as proportions (%) and continuous variables -as means. The distribution of the eGFR means across quartiles of the lipid profile parameters was carried out by one-way ANOVA analysis. Pearson or Spearman correlation coefficients were calculated for testing a linear relationship between eGFR and lipid profile parameters. We estimated collinearity and multicollinearity with variation inflation factors (VIF) for all lipid profile parameters. Linear regression models, both simple and multiple, were built between eGFR and covariates. The final stepwise forward linear regression model comprised the Apolipoprotein B variable as the main exposure. All covariates included in the model were tested for interaction. A linear trend of eGFR across the quartiles Q1–Q4 of the lipid parameters was performed with a logistic regression test of “departure from a linear trend”.

ROC analysis was employed to evaluate the predictive ability of the lipid profile parameters in early pre-CKD eGFR decrease and the validity of Apolipoprotein B based on logistic regression.

### 2.8. Participants

**Figure 2:**
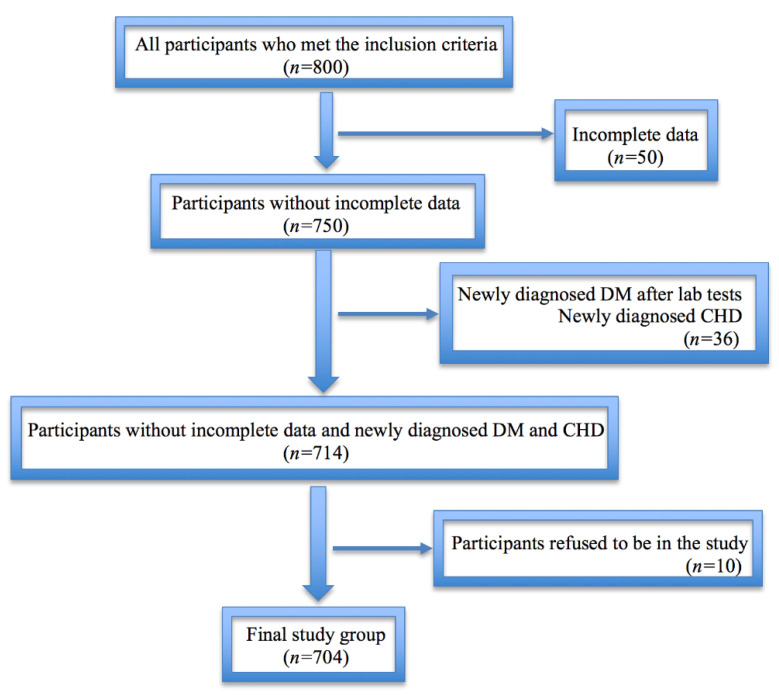
Flow chart showing participants’ selection process.

The flow chart of the participants’ selection is shown in Figure 2 below. Initially, 800 participants who met the inclusion criteria had been recruited into the study. After revising the IRC of every subject and their medical records, we have revealed 50 individuals with incomplete data and/or mistakenly missed comorbidities as exclusion criteria, such as coronary heart disease (CHD), thyroid disorders, and neoplasms. At the stage of laboratory and diagnostic tests, additional 36 cases had been detected as newly diagnosed diabetes mellitus (DM) and CHD (22 and 14, respectively). Further, ten subjects have left the study. Thus, the final database contained data of 704 participants of Kazakh ethnicity with grades 1–3 of essential hypertension free from diabetes, CVDs, thyroid dysfunction, CKD, acute kidney condition, nephrotic syndrome, neoplasm, mental disease, and those not being on a regular statin therapy less than six months prior the onset of the study. All the study participants had been under the care of primary care physicians for one to two decades, receiving antihypertensive treatment as well as lifestyle change advice with an uncertain level of adherence. None of them was on statin therapy at the moment of recruiting into the study.

## 3. Results

### 3.1. Descriptive Data

The baseline demographic and clinical characteristics of the study group are presented in Table 1. Out of 704 participants with an average age of 52.4 years old, 314 were males and 390 females. Mean eGFR was 91.01 mL/min per 1.73 m^2^. Eighty-two percent of the study participants follow healthy eating habits, while nobody follows unhealthy eating habits (1–4 points).

Table 2 presents the unadjusted distribution of eGFR among quartiles of lipid profile parameters. Higher Apolipoprotein B/A1 ratio, Apolipoprotein B, LDL-C, TG, and TC quartiles were associated with a lower level of eGFR. We did not find differences in the means of eGFR between quartiles of Apolipoprotein A1 and HDL-C.

The preliminary correlation test showed that a linear relationship of eGFR was observed only with Apolipoprotein B, LDL-C, and Apolipoprotein B/apoA1 ratio, which was in all cases negative and small (*r* = −0.23, −0.27 and −0.16, respectively). In contrast, Apolipoprotein A1, HDL-C and TG did not have a linear relationship with eGFR (*r* = −0.06, *r* = −0.06, and *ρ* = −0.045, respectively).

Multicollinearity test with VIF above five has restricted LDL-C, HDL-C, and TC variables from the inclusion to the model. Thus, the final linear regression model contained only Apolipoprotein-B out of lipid profile parameters.

**Figure 3:**
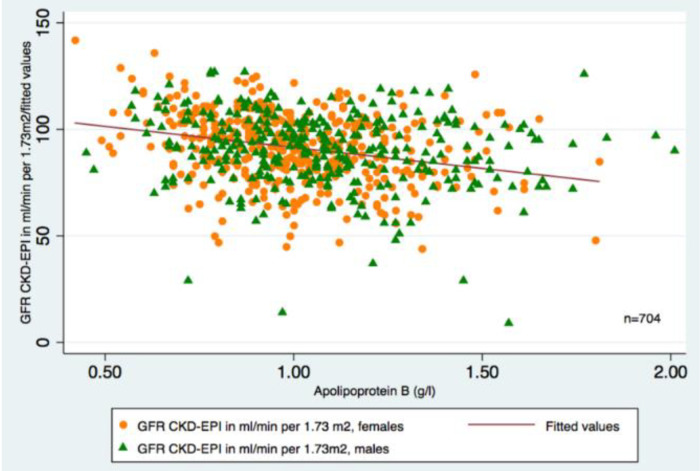
Scatterplots for a simple linear regression between eGFR and Apolipoprotein B ^ψ^. ^ψ^ Regression equation: eGFR = 101.95 − 10.70*Apolipoprotein B g/L.

Crude linear association between eGFR and Apolipoprotein B is displayed in Figure 3 and Table 3. Elevated across quartiles from Q1 to Q4, Apolipoprotein B was accompanied by statistically significant decreased eGFR. The higher the quartile of Apolipoprotein B, Apolipoprotein B/A1 and LDL-C, the lower the level of eGFR was observed.

Table 3 presents the final multivariable linear regression models for the association between eGFR and Apolipoprotein B. After adjusting for age, DQS, and income, the relationship between eGFR and Apolipoprotein B remains strong. The regression equation for the final multivariable model is: eGFR = 106.60 − 4.18*ApoB_Q2_ − 3.91*ApoB _Q3_ − 7.44*ApoB _Q4_ − 10.46*Age _40–49 yrs_ − 18.20*Age _50–59 yrs_ − 28.07*Age _60–69 yrs_ − 36.16*Age _≥ 70 yrs_ + 2.72*DQS _7/11_ + 0.93*Income _50–100,000 kzt_ − 6.43*Income _>100,000 kzt_.

The higher the quartile of Apolipoprotein B, the stronger the renal function’s negative impact has been observed. Age and income have a similar inverse effect on the eGFR. Aging by every ten years in the study participants has been accompanied by a 10 mL/min per 1.73 m^2^ decrease in eGFR on average from 10.46 mL/min per 1.73 m^2^ in the strata 40–49 years old to 36.16 mL/min per 1.73 m^2^ in participants older 70 years old. In contrast, DQS was positively associated with eGFR (the slope was 2.72 with a *p*-value of 0.045). Of the variability, 35.5% of eGFR is explained by its relationship with Apolipoprotein B (R^2^ = 35.5). Income above 100,000 KZT demonstrated an inverse linear association of Apolipoprotein B with eGFR (*p* = 0.028).

### 3.2. ROC Analysis of the Predictive Value of Apolipoprotein B in Pre-CKD eGFR Decrease

**Figure 4:**
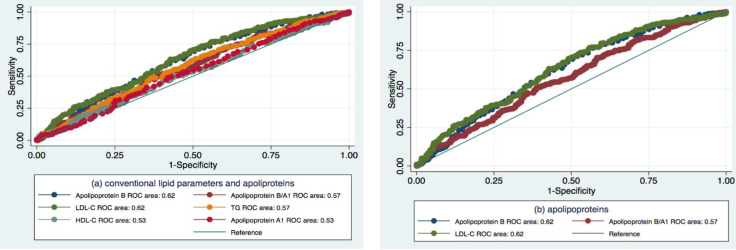
ROCs and AUCs of the lipid profile parameters (**a**,**b**) in the prediction of pre-CKD eGFR decrease.

We have performed ROC regression to compare the predictive accuracy of Apolipoprotein B with other lipid profile parameters. Apolipoprotein B (AUC = 0.62, SE = 0.02, 95%CI 0.57; 0.66) was very similar to LDL-C (AUC = 0.62, SE = 0.02, 95%CI 0.59; 0.67) (Figure 4). Sixty-two percent of the time, individuals with higher Apolipoprotein B levels will have a lower level of eGFR. Apolipoprotein A and HDL-C demonstrated weak predictive value (AUC = 0.53, SE = 0.02, 95%CI 0.49; 0.57 and AUC = 0.53, SE = 0.02, 95%CI 0.48; 0.57, respectively). Apolipoprotein B/A1 and TG showed low AUC as well (AUC = 0.57, SE = 0.02, 95%CI 0.52; 0.61 and AUC = 0.57, SE = 0.02, 95%CI 0.53; 0.62, respectively). All differences were statistically significant, *p* = 0.0007.

**Figure 5:**
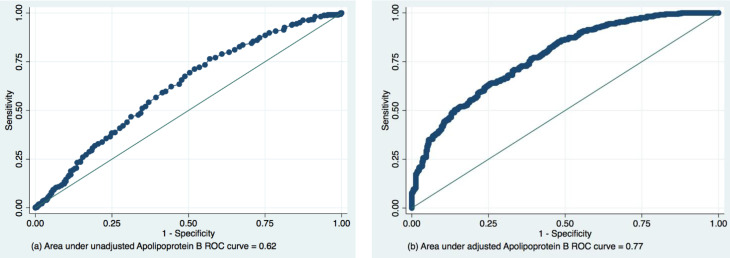
Unadjusted (**a**) and adjusted (**b**) ^ψ^ ROCs ^α^ and AUCs ^β^ for the predictive value of Apolipoprotein B in pre-CKD eGFR decrease ^ξ^. ^α^ Receiver operating characteristic; ^β^ Area under the curve; ^ψ^ Adjusted for age, DQS and income; ^ξ^ from logistic regression.

We have measured the global performance of Apolipoprotein B in early pre-CKD eGFR decrease by ROC based on a fitted logistic regression model. We assessed the area under the ROC curve before and after the adjustment for the potential confounding factors. Adjustment for age, DQS and income has strengthened the model’s predictive power in pre-CKD from 0.62 to 0.77 (Figure 5).

**Figure 6:**
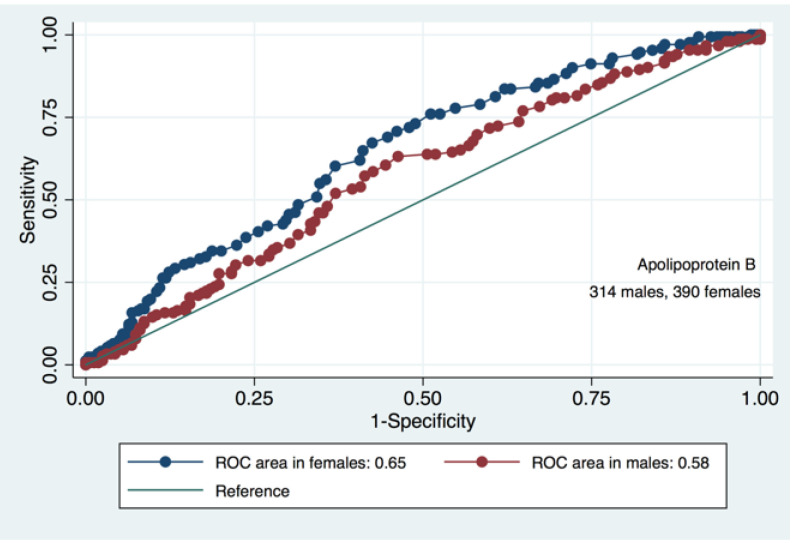
ROCs and AUCs for the predictive value of Apolipoprotein B in pre-CKD eGFR decrease in males and females.

We have found that diagnostic value of Apolipoprotein B for pre-CKD is greater in females compared to males (AUC = 0.65, SE + 0.02, 95%CI 0.60;0.70 and AUC = 0.58, SE = 0.03, 95%CI 0.51;0.64, respectively, *p* = 0.09) (Figure 6).

**Figure 7:**
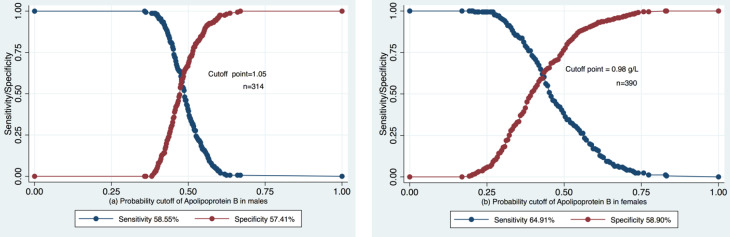
Cutoff values, sensitivity and specificity of Apolipoprotein B, stratified by gender (**a**,**b**).

We determined cutoffs of Apolipoprotein B separately for males and females (Figure 7). Both lipids displayed higher estimates in males compared to females. The optimal cutoff point of Apolipoprotein B in males was 1.05 g/L versus 0.98 g/L in females.

## 4. Discussion

Our study found a significant negative association between early kidney dysfunction, i.e., pre-CKD condition with G1 and G2 categories of eGFR [15] mitigation and Apolipoprotein B Kazakh hypertensives without comorbidities such as CVD, diabetes, CKD, acute kidney conditions, and nephrotic syndrome. We did not observe a linear relationship between Apolipoprotein A1 and GFR, HDL-C and eGFR, or TG and eGFR at all (*r* = −0.06; −0.06, and 0.045, respectively). Alongside, we have measured the predictive ability, optimal cutoff points, and validity of Apolipoprotein B in pre-CKD discrimination. We suppose that 1.05 is an optimal cutoff point of Apolipoprotein B for males (sensitivity 58.55%, specificity 57.41%) and 0.98 (sensitivity 64.91%, specificity 58.90%) for females in discriminating individuals with and without pre-CKD eGFR decline. To our knowledge, no previous studies have investigated cut-off points of Apolipoprotein B in pre-CKD. The observed difference could be a result of gender patterns in lipid metabolism and/or the impact of behavioral factors beyond the study’s scope.

So far, there is a paucity of scientific literature specifically relevant to the relationship between dyslipidemia and pre-CKD eGFR decline. Most studies have been conducted on populations with obvious CKD, including patients with advanced stages and hemodialysis due to diabetes or CVD where eGFR dramatic reduction was strongly and consistently related to CKD progression. In contrast, Diabetes, CKD, and CVD were exclusion criteria in our study, and all participants had eGFR above 60 mL/min per 1.73 m^2^.

A crucial position of a lipid profile in CVD prediction has been reflected in the numerous current clinical guidelines with established even target treatment goals of LDL-C for patients with different degrees of CVR. There is extensive proof for the causative position of LDL-C in CVD development [21,22]. Apolipoprotein B, as a structural component of LDL-C, VLDL-C, and TG, plays a key role in early atherosclerosis [23]. The high predictive power of Apolipoprotein B and Apolipoprotein B/A1 ratio in CVD and early pre-disease condition, insulin resistance, diabetes, and metabolic syndrome (MS), have been highlighted in several studies [12,24,25,26,27]. Simultaneously, some authors reported conflicting data on the relationship between HDL-C and CVR despite a substantial body of evidence for HDL-C’s preventive effect against CVD [28,29]. The general scientific perspective considers HDL-C and its principal constituent Apolipoprotein A1 as inversely related to CVD and atherosclerosis development [30,31,32]. 

CKD enormously increases CVR in patients. Reduced GFR is a major predictor of atherosclerotic CVD (ASCVD) [33,34,35,36,37]. Even mild renal insufficiency is associated with a four-fold risk of cardiovascular death [38]. Hypertension and diabetes are the most common causes of CKD. However, CVR is frequently underestimated in CKD. In this regard, dyslipidemia, i.e., Apolipoprotein B, appears to unambiguously contribute to a more accurate CVD risk prediction and evaluation [39].

Total cholesterol (TC) and LDL-C were found to be independent risk factors of end-stage renal disease (ESRD) in diabetes type 1 and type 2 [40,41,42]. Accumulating research highlights TG as the main CVD and CKD predictor in diabetes [43,44]. Hypertriglyceridemia leads to both albuminuria and endothelial damage [45]. Only a few publications discuss the relationship between the lipid profile parameters and kidney dysfunction, which are partly controversial. The role of dyslipidemia in the development of CKD is still not fully clear [46]. In CKD-free individuals, dyslipidemia can predict proteinuria and onset of CKD [47]. Tozawa et al. reported high triglyceride in females and low HDL-C levels in males and increased LDL-C as potential predictors of the decline of kidney function in patients without proteinuria [39,48]. Although analysis of ARIC (the Atherosclerosis Risk in Communities) study and the Third National Health and Nutrition Examination Survey (NHANES III) have revealed no association between Apolipoprotein B and eGFR, Apolipoprotein A1 and Apolipoprotein B/A1 ratio were significantly inversely associated with eGFR. Both samples included patients with different stages of CKD [10].

In comparison, a recent cross-sectional Chinese study with a sample size of 8322 was derived from the China Health and Nutrition Survey (CHNS) reported a strong positive association between Apolipoprotein B and stages of CKD through eGFR even after adjusting for potential confounding factors [11]. Our findings are in agreement with the high predictive ability of Apolipoprotein B in terms of pre-CKD also. However, what remains unclear is why HDL-C has not demonstrated its association with pre-CKD eGFR decline in non-diabetic hypertensives in our study, as previously reported in diabetic patients [49]. This could be explained by the early pre-disease state of eGFR or possible initial lower levels of HDL-C and Apolipoprotein A1 in Kazakhs due to regulatory genetic patterns of lipid metabolism. Further research focused on the relationship between clinical CKD and HDL-C and/or Apolipoprotein A1 could confirm our preliminary findings and highlight their role in CKD patients.

Zhang et al. have demonstrated no association between TC, TG, LDL-C, and Apolipoprotein B with eGFR in patients with CKD. Only HDL-C was significantly positively associated with renal dysfunction [50]. Seok-Hyung Kim et al. detected Apolipoprotein B/A1 ratio’s predictive ability for coronary artery calcification only in patients with normal kidney function [51]. Dyslipidemia deteriorates and contributes to kidney dysfunction. Studies suggest that CKD resulted in renal microvasculature and renal parenchymal dysfunction with further foam cell acceleration, boosting oxidized LDL synthesis [52,53,54].

In the present study, we have supposed Apolipoprotein B in the early recognition of pre-CKD in non-diabetic individuals. TGs, HDL-C, and Apolipoprotein A1 were not found to be related to eGFR in this regard. Age, DQS, and income appear to significantly impact the association between Apolipoprotein B and eGFR. These changes likely predict early renal dysfunction in the Kazakh population. Further large-scale and perhaps genetic research will bring more evidence at that point and clarify the role of age, DQS, and income and the lack of relationship of eGFR with HDL-C and Apolipoprotein A1.

### Limitations

Certain circumstances restricted the validity of our research. Firstly, the study population includes hypertensives only, which shortens the generalizability of the results. Secondly, as the current study is a part of a larger project and establishing albuminuria was beyond the scope of the primary research, laboratory tests did not count urine albuminuria, which might be a potential source for misclassification of CKD status in the study participants.

## 5. Conclusions

Apolipoprotein B is strongly inversely associated with eGFR in the early pre-CKD condition in Kazakh hypertensives. Age, DQS, and income significantly fitted the final model increasing the predictive ability of Apolipoprotein B from 0.62 to 0.77. The optimal cutoff point of Apolipoprotein B for males is 1.05 g/L (sensitivity 58.55%, specificity 57.41%) and for females is 0.98 g/L (sensitivity 64.91%, specificity 58.90%).

## Figures and Tables

**Table 1:** Baseline demographic and clinical characteristics of the study population.

Variables	Absolute Number	Proportion or Mean/SD *
*n* of the study population	704	100%
Gender, male	314	44.6
Age (y):		52.4/10.6
<39	71	10.1
40–49	216	30.7
50–59	232	33.0
60–69	154	21.9
>70	31	4.4
MS	384	54.6
Education, university or higher	219	31.1
Income, KZT/capita/month:		
<50,000	329	46.7
50,000–100,000	351	49.9
>100,000	24	3.4
Smoking, current	132	18.8
Alcohol consumption, current	293	41.6
Gym class	73	10.4
Hereditary for hypertension	408	58.3
Hereditary for CHD	158	22.4
GTT, abnormal	120	22.2
DQS less than 7	126	17.9
eGFR by CKD-EPI, mL/min per 1.73 m^2^, mean	704	91.01/16.86
Apolipoprotein B, g/L	704	1.04/0.26
Apolipoprotein A1, g/L	704	1.46/0.25
Apolipoprotein B/A1 ratio	704	0.73/0.22
HDL-C, mmol/L	704	1.44/0.42
LDL-C, mmol/L	704	3.48/0.90
Triglycerides, mmol/L	704	1.53/3.9
Total cholesterol, mmol/L	704	5.03/0.94

CHD—Coronary heart disease; GTT—Glucose tolerance test; DQS—Diet Questionnaire Score. * Data are presented as a proportion for binary and categorical variables and mean/SD (standard deviation) for continuous variables.

**Table 2:** Unadjusted distribution of eGFR across quartiles of lipid profile parameters based on the one-way ANOVA analysis.

Parameters	Quartiles				*p*-Value	Barlett’s Test
	Q1	Q2	Q3	Q4		
Apolipoprotein B/A1	<0.56	0.57–0.70	0.71–0.84	>0.84		
eGFR, mL/min per 1.73 m^2^	95.11	90.47	90.14	88.33	0.0013 0.003 *	0.56
Apolipoprotein B	<0.85	0.86–1.0	1.01–1.18	>1.18		
eGFR, mL/min per 1.73 m^2^	96.89	91.08	89.75	86.16	0.001 0.001 *	0.001
Apolipoprotein A1	<1.28	1.29–1.43	1.44–1.62	>1.62		
eGFR, mL/min per 1.73 m^2^	92.45	91.73	90.39	89.54	0.36 0.22 *	0.67
LDL-C	<2.84	2.85–3.42	3.43–4.08	>4.08		
eGFR, mL/min per 1.73 m^2^	98.82	90.90	88.90	85.63	0.001 0.001 *	0.068
HDL-C	<1.14	1.15–1.38	1.39–1.67	>1.67		
eGFR, mL/min per 1.73 m^2^	91.52	92.24	90.17	90.11	0.57 0.22 *	0.54
TG	<0.85	0.85–1.13	1.14–1.69	>1.69		
eGFR, mL/min per 1.73 m^2^	95.20	90.51	89.86	88.59	0.0015 0.002 *	0.045
TC	<4.32	4.33–4.94	4.95–5.62	>5.62		
eGFR, mL/min per 1.73 m^2^	97.67	89.99	89.02	87.45	0.001 0.001 *	0.018

* *p* for linear trend was obtained from binary logistic regression.

**Table 3:** Crude and adjusted linear multivariable-adjusted regression models for the association between mean eGFR and quartiles of Apolipoprotein B.

Means of eGFR (mL/min per 1.73 m^2^) Across Quartiles of Apolipoprotein B (g/L)				Adjusted for:	Models	Number of Observations
Q1 (<0.85)	Q2 (0.86/1.0)	Q3 (1.01/1.18)	Q4 (>1.18)			
Baseline	−5.81 ^ψ^ (−9.24; −2.39) *p* = 0.001	−7.14 ^ψ^ (−10.57; −3.71)	−10.73 ^ψ,^^α^ (−14.21; −7.26)	Crude	1	704
Baseline	−3.79 ^ξ^ (−6.67; −0.92)	−3.69 ^ξ^ (−6.58; −0.80)	−7.53 ^ψ^ (−10.46; −4.61)	Age	2	704
Baseline	−3.94 ^φ^ (−6.81; −1.07)	−3.84 ^γ^ (−6.74; −0.95)	−7.46 ^ψ^ (−10.38; −4.54)	Age + DQS	3	704
Baseline	−4.18 ^η^ (−7.04; −1.31)	−3.91 ^ε^ (−6.80; −1.02)	−7.44 ^ψ^ (−10.35; −4.52)	Age + DQS + income	4	704

Baseline related to the mean eGFR at Q1 of Apolipoprotein B in every model. ^ψ^ *p* < 0.001; ^ξ^ *p* < 0.01; ^η^ *p* = 0.004; ^φ^ *p* = 0.007; ^ε^ *p* = 0.008; ^γ^ *p* = 0.009; ^α^ *p*-value for linear trend < 0.001.

## Data Availability

The data presented in this study are available on request from the corresponding author.
